# S11-2: Reaching and recruiting young people experiencing homelessness for physical activity interventions: Challenges, opportunities, and recommendations

**DOI:** 10.1093/eurpub/ckae114.247

**Published:** 2024-09-26

**Authors:** Jennifer Thomas, Diane Crone, Nicola Bowes, Katie Thirlaway, Robert W Meyers, Kelly A Mackintosh

**Affiliations:** Applied Sports, Technology, Exercise and Medicine (A-STEM) Research Centre, Swansea University, Wales, UK; School of Sport and Health Sciences, Cardiff Metropolitan University, Wales, UK; School of Sport and Health Sciences, Cardiff Metropolitan University, Wales, UK; School of Sport and Health Sciences, Cardiff Metropolitan University, Wales, UK; School of Sport and Health Sciences, Cardiff Metropolitan University, Wales, UK; Applied Sports, Technology, Exercise and Medicine (A-STEM) Research Centre, Swansea University, Wales, UK

## Abstract

**Purpose:**

There is a growing body of evidence to suggest that physical activity-based interventions may be effective for engagement, psychological outcomes, and behaviour change of young people experiencing homelessness (YPEH). Nonetheless, challenges associated with reaching and recruiting this marginalised population are frequently reported owing to social isolation, lack of trust, and a high prevalence of poor mental health. This study sought to explore common barriers to recruitment, and identify potential strategies through which these may be overcome.

**Methods:**

Three 8-week physical activity interventions were delivered to groups of between 4 and 12 YPEH. Following each intervention, qualitative data was collected through a series of interviews with implementers (sports coaches; n = 5) and support staff (n = 3), and three focus groups with participants (n = 17). Documented feedback, such as informal correspondence with staff, were used to provide further insights. Data were transcribed verbatim and analysed using a mixed inductive/deductive thematic approach to identify multi-level challenges and opportunities, and comprehensively address the research aims.

**Results:**

Multiple barriers and opportunities to the reach and recruitment of YPEH were identified across organisational, individual, and intervention levels. Challenges included limited staffing and organisational resources, negative experiences of participants, and sub-optimal intervention design and materials. Enablers included co-production with stakeholders, organisational staff champions, participant incentives, appropriate materials, and an active outreach recruitment strategy.

**Conclusions:**

Overcoming challenges associated with reaching and recruiting marginalised groups for physical activity interventions requires consideration of multi-level barriers from the early stages of intervention development. While the enablers identified are concurrent with those reported across wider literature, future research should focus on how these can be embedded in a co-productive manner involving all stakeholders, throughout the research process.

**Support/Funding Source:**

Knowledge Economy Skills Scholarships (KESS 2); European Social Research Council Postdoctoral Fellowship Programme.

